# Workshop of the Task Force on Migration and Mental Health

**DOI:** 10.1192/j.eurpsy.2025.197

**Published:** 2025-08-26

**Authors:** S. -.- Bäärnhielm

**Affiliations:** Clinical Neuroscience, Karolinska Institutet, Stockholm, Sweden

## Abstract

**Abstract: Introduction:**

Sweden has a long history as a host country for refugees. In recent years there has been a shift from being one of the most generous host countries in Europe to one of the most restrictive. In Sweden, refugees with a residence permit have a full right to care, but asylum seekers and undocumented migrants only have a right to care that cannot be deferred. Despite relatively good formal access to mental health care, and so far, the availability of free language interpreters, refugees face barriers to and within mental health care.

**Aims:**

To give a brief overview of the current challenges for mental health care in Sweden and the work on solutions for people on the move.

**Research methods:**

Ongoing research and clinical development are summarised in parallel with the identification of challenges.

**Findings:**

A complex picture of the development and challenges of mental health care for people on the move in a situation of increasing social pressure on refugees is described.

**Conclusions:**

There is a need for equal treatment of people on the move without discrimination and exclusion.

**Disclosure of Interest:**

None Declared

